# Criminal Lunacy

**Published:** 1856-02

**Authors:** 


### EDITORIAL DEPARTMENT.

---

*Criminal Lunacy.*—In a conversation held with a lawyer of large experience in criminal law, he expressed a regret that the medical profession should have so run wild on the questions of legal insanity, as he asserted they have. In all cases where the plea of insanity is made, the counsel for the defence may rely with tolerable certainty on sufficient medical evidence to acquit the prisoner. And it is undoubtedly true that this defence has been so freely and successfully used in this country, as to remove one of the greatest protections to personal safety, and to encourage the unlawful shedding of blood.

It has now become a question for the profession to decide, whether they will sustain the present lax doctrines of legal responsibility; or whether, with a view partly to their own credit, and to the good of the community, they shall assume the difficult duty of acting as mediators between the rigor of the law and the truly irresponsible criminal, and at the same time of protecting society by a fearless discrimination between real and pretended insanity.

The faults of medical evidence may be assigned to a variety of causes. The witness may be a believer in the materialistic doctrines of phrenology. If so, he naturally accounts for a murder on the supposition of an undue development or excitement of the organ of Destructiveness. The prisoner, he says, is the victim of an inherent fault in the construction of his brain—he is no more responsible for it than is the cripple for his club-foot. To a person indulging such a belief, a decision in favor of the insanity of the prisoner is a logical necessity; and were he called on to testify in a case of theft, even, it would probably be the same.

Again; the witness may be one of that class of minds who deny that any one is entirely sane, who look upon the human mind as such an extremely imperfect instrument, that all men are liable to sudden perversions of the sense of right and wrong, resulting in acts which *would be criminal* if they were the acts of a truly sound mind. They argue that no one in the sober possession of his senses, will commit a murder, therefore all murderers are insane. The argument has truth enough to be dangerous, but we have only to extend it to minor crimes, to theft, adultery, and even those crimes requiring a clear head and acute mind (such as forgery, swindling, &c.,) to perceive that it is an argument equally applicable to them, and of course subversive of all criminal law.

Or, another medical witness may have adopted the notion of which Dr. Thomas Mayo in the leading exponent, viz., that the immaterial mind itself may be diseased. Now if this be true, a murder may be the result of such an immaterial disease, unaccompanied by any cerebral lesion or symptoms. We cannot estimate the action of an immateriality—it may impel a man to murder while the face is calm, the reason undisturbed, and every function cognizable to the medical observer is in its normal condition. A single glance is sufficient to discover the danger of this doctrine. We assume a disease of a thing—or a nothing—the very existence of which is insusceptible of demonstration. We do not where it is or what it is; we do not know how disease may act upon it; but if we believe it capable of being diseased, we have a right to assume, in any case, that it is so; and that in the absence of any of the usual and recognized symptoms of insanity. But so far as an immateriality can be demonstrated, De Boismont has proved that the mind is—like the king in the game of chess—inviolable—that however feebly, imperfectly, or erroneously its instrument, the brain, may act, itself remains in eternal health.

Still another common metaphysical error, in medical witnesses, has a contrary tendency upon the prisoner's interests. It is the fashion, with some men, to believe that insanity and moral depravity are identical. This is the converse of the materialistic doctrine already alluded to. The phrenologist would make all sin and insanity identical, but would look upon the sin as the offspring of the insanity, and upon both as derived from an abnormal brain. On the other hand the followers of Heinroth or of Ideler, the German metaphysicians, and their numerous disciples on this side of the Atlantic, would reverse the operation. Considering insanity as identical with spiritual sin, they would make the former dependent on the latter, and would punish it as in itself a crime, at any rate a manifestation of a strong criminal tendency.

Now it is the duty, and, we are happy to believe, the habit, of the law to look to the consequences of a belief before acknowledging its truth. The decisions of our courts in questions involving matters of belief have ever constituted a strong barrier against radicalism. Looking to this principle of action, it seems to us that it would not be a very objectionable plan to declare any witness holding these forms of belief incompetent. Such creeds are entirely inconsistent with all the theories of law, and its practice ought not to be influenced by them.

But other causes not less theoretical too often temper the testimony of the medical witness against the ends of the law, and sometimes, but rarely, do injustice to the prisoner.

In the first place we feel a natural aversion to say, positively and definitely, that the prisoner is not insane within the meaning of the law. His life, perhaps, rests upon our single word, and the responsibility of thus taking it from him is, indeed, a heavy one. But it is one of those duties which every man must assume for the common protection of all, and no personal faint-heartedness should prevent our utterance of our honest opinion, guiding and forming that opinion by looking at the question as an abstract one, without reference to consequences.

Secondly; the borders of sanity so shade off into those of insanity, that it is often difficult to decide where the line should be drawn. Naturally the witness would sooner err on the side of mercy than that of judgment. But it is not of this that jurists, in their censures of medical witnesses, complain. It is that they allow themselves so often to mistake a fit of passion for one of insanity. The man A, for instance, suffers a deep wrong at the hands of B; one for which the law provides no competent redress. His wife, perhaps, has been seduced, and looking for justice he finds that none can be obtained. "Here," he says, "I must right myself. B is a criminal who does not deserve to live. The law does not defend me. When I surrendered the control of my actions to the law, it was with the understanding that it should shield me from wrong. It fails to do sc, the contract is dissolved, and I am now at liberty to be a law unto myself." Acting under this entirely rational, though mistaken idea, A takes the life of his injurer B. Is the act an insane one? On the contrary, it is a mere error of reason, such as, in less important matters, the sanest man may any day commit.

The popular tenderness for such crimes is the result of a — so to speak — metaphysical blunder. To be rational does not, as some would seem to infer, imply correctness of reason. The very fact that we do reason implies a doubt that we are not sure what is right, and must study to find the proper course to be pursued. God is not a rational being — to reason implies imperfection. No man has a right to make to himself a "higher law" than the law of the land. The right to occasional violations of laws of seeming harshness once admitted, and every man is at liberty to choose what he will or what he will not obey. There is not a month in the year that some criminal does not escape justice in cases of this kind, through the unwillingness of medical witnesses to point out to jurors the real distinction between an error of reason and an insanity.

But another, and a more real difficulty exists, which, so long as the present law or ruling of the courts obtains, must continue to embarrass the medical witness, and often to pervert the ends of justice. His evidence must be, given so as to mean one of two things; either the prisoner was, or was not capable of discriminating between right and wrong at the time of the crime charged. The most competent medical witness must frequently fail to answer this question properly, for the reason that both the affirmative and the negative may be wrong. Insanity does not always weigh a pound, or measure a pint. It has its degrees and conditions, shading off from a mere flightiness of reason, to the darkest form of homicidal mania. Constitutionally, some men yield more readily to erratic influences than others. Now, if there are degrees of insanity, there are degrees of criminality in the insane,—from the innocent but fatal impulse which led the sister of Charles Lamb to stab to the heart the mother whom she tenderly loved, to the cunning, malicious act of him who purposely, almost sanely, allows himself to be subjected to a homicidal impulse which he might, if he chose, resist, and would resist if he feared punishment.

We mean to endorse the doctrine lately advanced by one of the most competent British writers on legal insanity—Dr. J. C. Bucknill—that "degrees of insanity exist for which exemption from punishment cannot be claimed." "It is not," he says, "and never has been, the law of this country, that all the pale and faint shades of mental disorder should shield the doer of a crime from the consequences of his act."

Take, for instance, the recent religious murder at New Haven. Doubtless the minds of the actors in that fearful tragedy were disordered, but can a belief in which hundreds of people participate, be called an insanity? If so, we must deduct from our list of sane men all the hundreds and thousands of our acquaintances who believe in spirit-rappings, mesmerism, or other humbugs.

In asserting that insanity should not always shield the doer of an act from its consequences, Dr. Bucknill means simply that a very wholesome restraint upon the acts of the partially insane may be exercised, by holding up a punishment for them. Of course where any ascertained and proven degree of insanity exists, the criminal should not suffer death, but some milder punishment, combining with it an effort for his real good and benefit. It is to supply this want in the laws governing questions of insanity, that Dr. Forb es Winslow has proposed that the jury should have power to return a verdic in three forms, viz., "guilty;" "not guilty on the ground of insanity;" and, thirdly, "guilty, with a recommendation to mercy on account of presumable insanity." Such a measure would accommodate the really large class of semi-insane criminals, and would leave to the medical witness the task of deciding only whether or no a man was rational enough to be a criminal, and whether he was criminal enough to be hung.

